# A rare case of skin blistering and esophageal stenosis in the course of epidermolysis bullosa - case report and literature review

**DOI:** 10.1186/s12876-018-0771-5

**Published:** 2018-04-13

**Authors:** Agata Michalak, Halina Cichoż-Lach, Beata Prozorow-Król, Leszek Buk, Monika Dzida

**Affiliations:** 1 Department of Gastroenterology with Endoscopy Unit, Medical University of Lublin, Jaczewski Str, Lublin, 820-954 Poland; 2 Department of Radiology and Nuclear Medicine, Medical University of Lublin, Jaczewski Str, Lublin, 820-954 Poland

**Keywords:** Epidermolysis bullosa, Esophageal stricture, Dysphagia, Endoscopic dilatation

## Abstract

**Background:**

Epidermolysis bullosa (EB) constitutes a heterogenous group of rare multisystem genetically transmitted disorders comprising several blistering muco-cutaneous diseases with a monogenic basis and either autosomal dominant or autosomal recessive mode of inheritance. EB manifestation is not only limited to the skin. Systemic signs might involve the nose, ear, eye, genitourinary tract and upper gastrointestinal tract. The presence of particular symptoms is directly determined by a type of altered skin protein. Gastrointestinal manifestation of EB is most commonly reflected by esophageal stenosis due to recurrent esophageal blistering, followed by consequent scarring.

**Case presentation:**

Here we present a case of a man with dystrophic EB and dysphagia, skin blistering, joints contractures and missing nails. To our knowledge, the presented man is the oldest one diagnosed with EB living in Poland.

**Conclusions:**

Management of an esophageal stricture in such circumstances is based on endoscopic dilatation. However, in most severe cases, placement of a gastrostomy tube is required. Despite great advances in medicine, a targeted therapy in the course of EB has not been established yet.

**Electronic supplementary material:**

The online version of this article (10.1186/s12876-018-0771-5) contains supplementary material, which is available to authorized users.

## Background

**Fig. 1:**
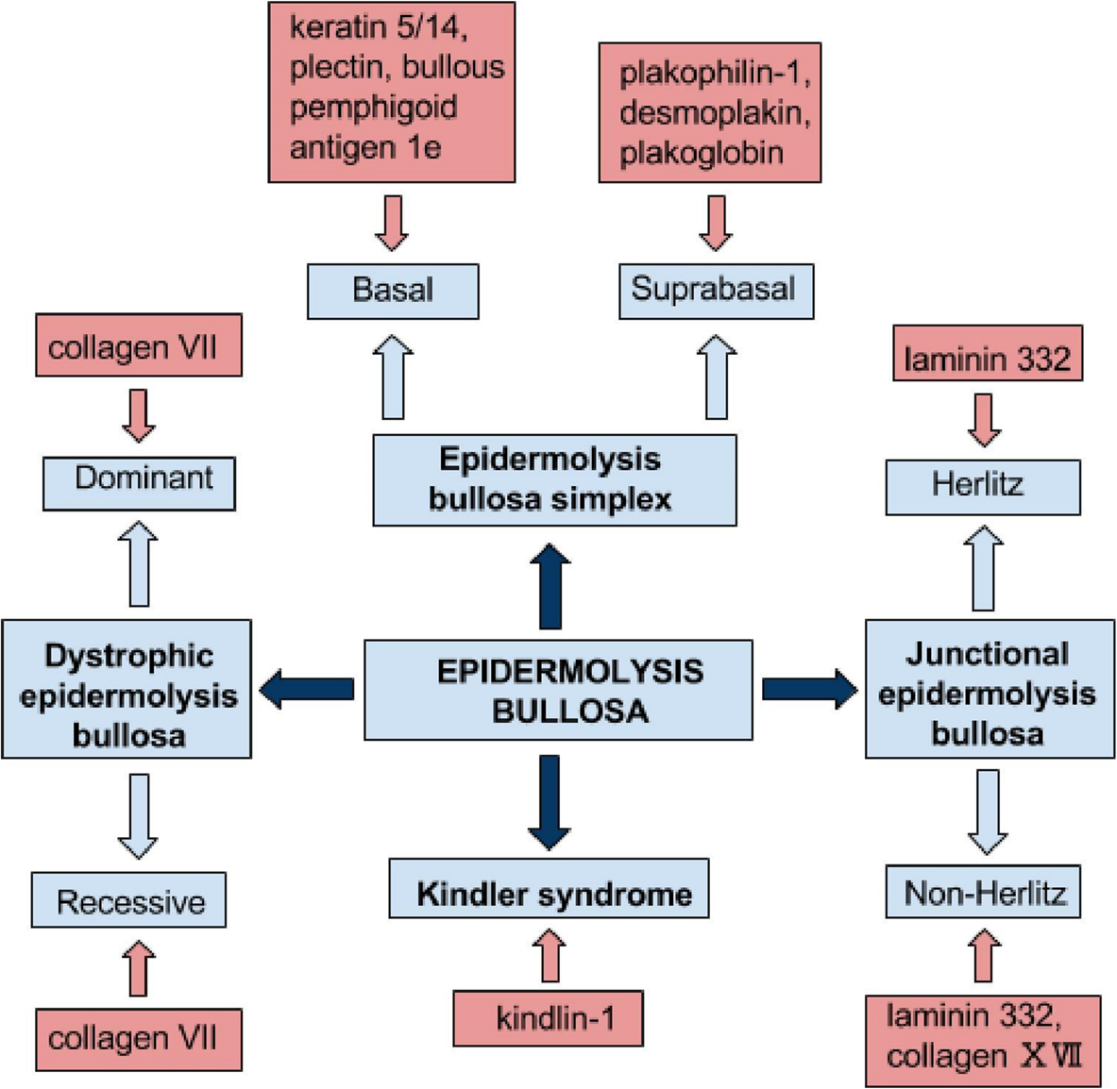
Types of epidermolysis bullosa with involved skin proteins

Epidermolysis bullosa (EB) is a broad entity which encompasses several types of genetically transmitted blistering disorders: EB simplex (EBS), junctional EB (JEB), dystrophic EB (DEB) and Kindler syndrome (KS). The division is mainly based on the level of skin cleavage. Extensive skin blistering as a result of minimal mechanical trauma is a key feature in the course of EB. Degeneration of particular types of skin proteins determines the presence of extracutaneous manifestation, whose range varies among EB subtypes [1, 2] (Fig. 1).

## Case presentation

**Fig. 2:**
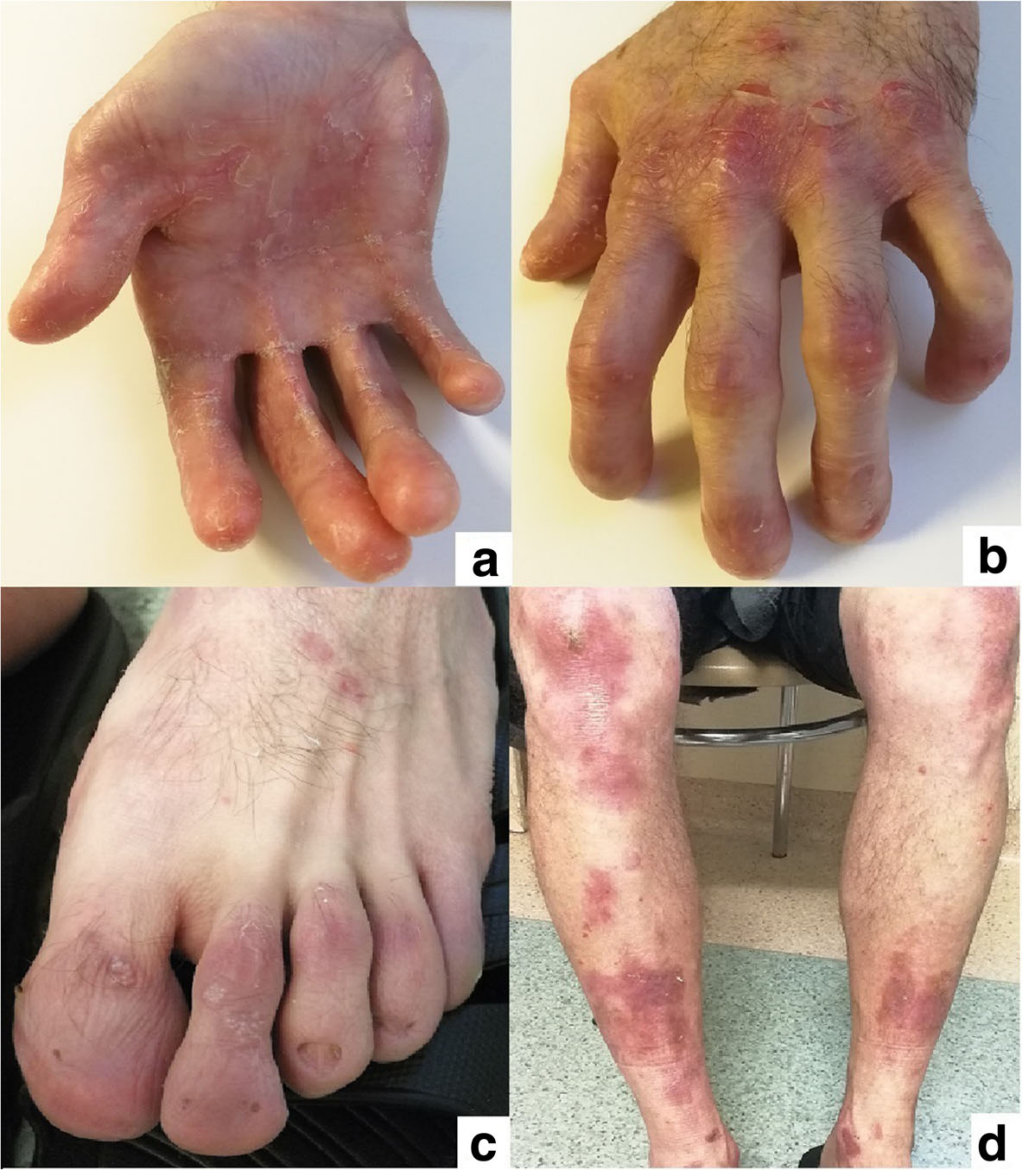
Contractures in hand joints and crust formation (**a**), blisters (**b**), loss of a finger and toenails (**b** and **c**), skin reddening (**d**)

**Fig. 3:**
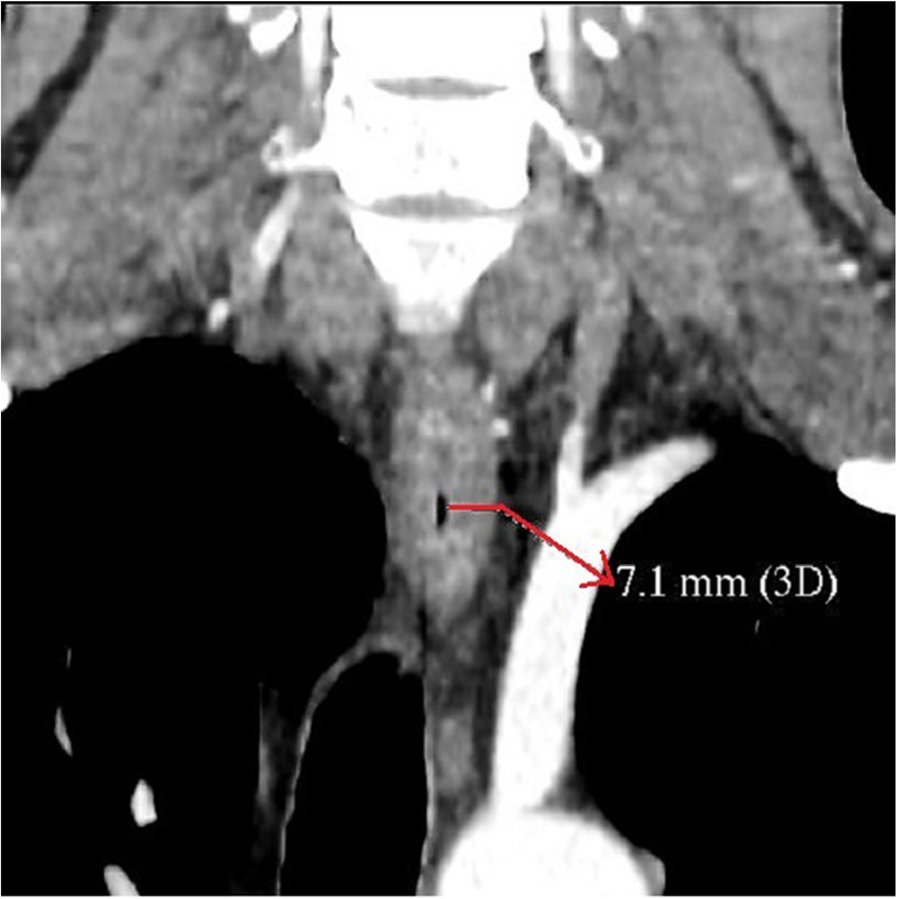
A thickening of the esophageal wall in a CT scan

**Fig. 4:**
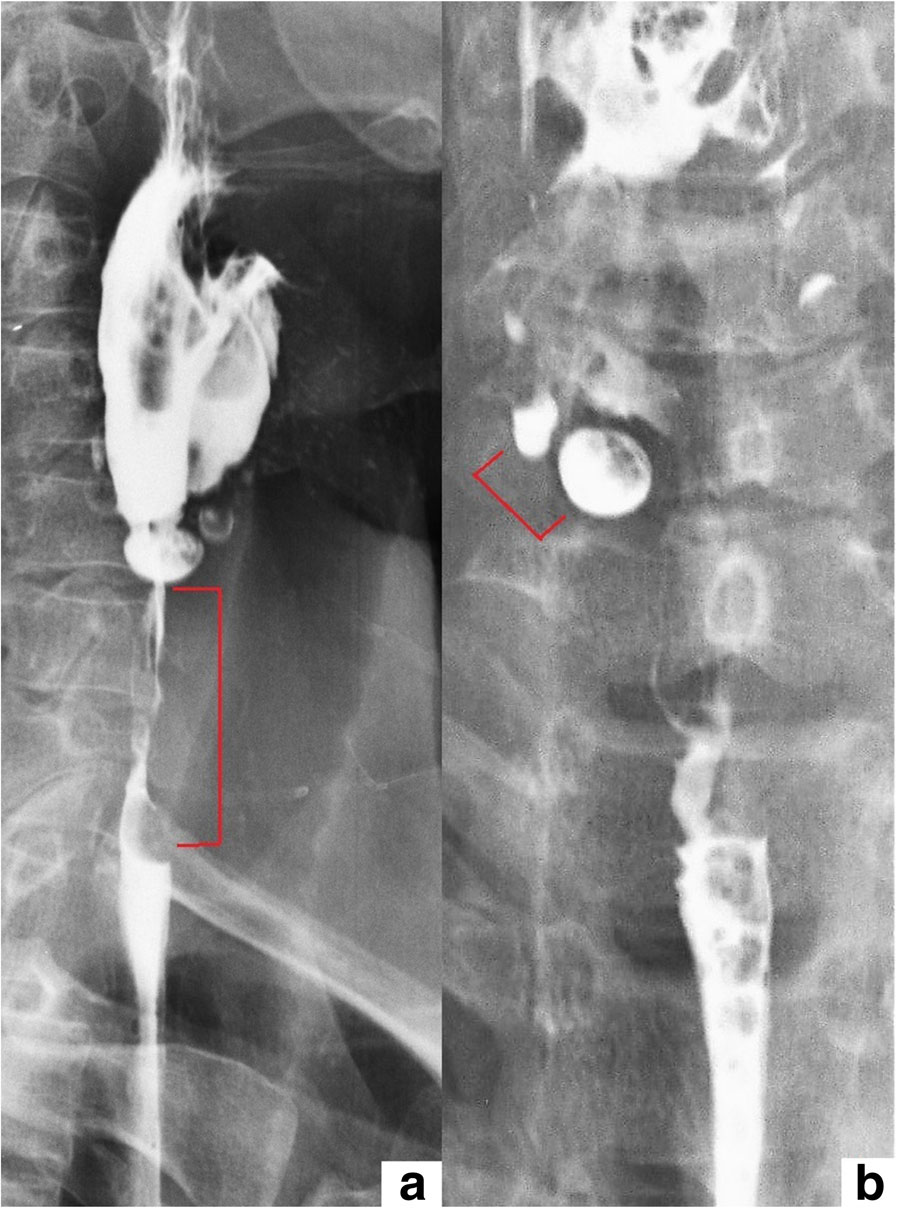
A narrowing of upper esophageal lumen (red line in picture **a**) and two esophageal diverticula (red line in picture **b**) in a barium swallow test

A fourty-year-old man with DEB diagnosed at the age of eight was admitted to the department of gastroenterology because of the dysphagia for two previous months. The diagnosis of DEB was established due to the presence of single blisters on the whole body since the sixth month of life. His sister was also diagnosed with DEB and had similar symptoms of the disease. To our knowledge, the presented patient and his sister are the oldest diagnosed with EB living in Poland. At the age of four the patient started experiencing heartburn occasionally. Five years later dysphagia appeared for the first time. It was an episodal and periodical ailment. He reported a deterioration of dysphagia at the age of nineteen; he mostly consumed liquids and soft consistency meals during that time. Nonetheless, the patient admitted that this esophageal discomfort still was not a constant one and there were time intervals without this ailment. In the past there were also episodes of mild esophageal bleeding. The only one endoscopic esophageal dilatation in this patient took place in 1997; a stenosis was located then approximately 18 cm from incisors. The performed procedure ameliorated swallowing difficulty. A barium swallow test obtained one year after the endoscopic dilatation of the esophagus also revealed esophageal constriction on the same level. In 2014 the patient was diagnosed because of hematochezia and pain in hypogastrium. Tissue samples obtained in colonoscopy revealed the presence of nonspecific inflammatory infiltration in the ascending colon and terminal part of the ileum. Interestingly, 3 years ago he complained of hemoptysis and there was a suspicion of bleeding to pulmonary alveoli in the course of DEB. However, a CT scan did not confirm bleeding. On admission to our department the patient was complaining of painful swallowing of solids. Two months earlier he was diagnosed in the cardiology unit because of the chest pain and elevated level of troponin I. An electrocardiogram did not show any abnormalities. The patient refused to undergo coronarography and no more cardiological diagnostic procedures were performed Additional file 1. On admission to our unit he did not complain of the chest pain. On physical examination he appeared comfortable, afebrile with pulse 90 beats per minute, blood pressure 125/90 mmHg, respiratory rate 19 per minute and the body mass index (BMI) was 24.7 kg/m^2^. The patient presented blisters, skin reddening and crust formation on the upper and lower limbs. There were also contractures and disabled movement in his hand joints together with a loss of a finger and toenails (Fig. 2). The apex of the tongue and left palatine arch were covered by superficial ulcerations. During his hospital stay, performed laboratory tests did not reveal any abnormalities. A CT scan of the chest and abdomen showed a thickening of the esophageal wall at maximum to 7 mm on the level from the fourth cervical vertebra to the fourth thoracic vertebra (Fig. 3). A probe of gastroscopy under sedation with benzodiazepine failed due to an esophageal stenosis. An attempt of examination with paediatric endoscope was also unsuccessful. A barium swallow test revealed a narrowing of upper esophageal lumen to 7 mm along the length of 4 cm together with two diverticula on the right side not emptying of contrast. During swallowing other two diverticula appeared which were emptying of contrast (Fig. 4). A barium swallow test also showed a noticeable weakening of the esophageal mucous membrane. After the performed investigation the patient was qualified to endoscopic dilatation of esophageal stenosis and endoscopic management of diverticula. However, he did not agree to undergo this procedure during current hospital stay. In our unit the patient was treated with proton pump inhibitor (PPI) and prokinetic drugs administered intravenously, which caused an amelioration of esophageal discomfort. He was discharged in a good general condition with a recommendation of a diet based on soft consistency meals, oral PPI and prokinetic drugs administration and the next follow-up in a month.

## Discussion and conclusions

In the 1886 German dermatologist Heinrich Koebner formed an idea of EB, a heterogenous group of genetically transmitted disorders comprising several blistering muco-cutaneous diseases with a monogenic basis and either autosomal dominant or autosomal recessive mode of inheritance. EBS is the most common form in western countries and in general has less severe skin lesions in comparison to JEB or DEB. The herlitz subtype of JEB is less prevalent than the nonherlitz JEB, but both might involve enamel hypoplasia. Skin scarring is a crucial symptom of the herlitz JEB subtype. What is more, mucosal surfaces of the esophagus, upper airway and cornea might also be affected. On the other hand, extracutaneous manifestation is not typical for the nonherlitz JEB. Dominant form of DEB is associated with the formation of skin blisters at birth [3, 4]. Recurrent involvement of the esophagus with subsequent scarring and stenosis can be observed among these patients. Recessive form of DEB is the most severe type of EB and finally leads to disfiguring skin scars, hand and foot deformities, growth retardation and failure to thrive. In addition to skin blistering, photosensitivity and skin pigmentation are characteristic features of KS. EB might involve extra-cutaneous manifestation, leading to severe complications in the eye, nose, oral cavity, ear, larynx and upper respiratory tract. Genitourinary complications (scarring of the glans penis or vaginal vestibule, urethral strictures leading to hydroureter and hydronephrosis) together with musculoskeletal involvement reflected by joints contractures, muscular dystrophy and pseudosyndactyly due to extensive blistering and scarring in DEB are also possible [5–7]. Anemia, usually present in patients with JEB and recessive DEB, heart involvement caused by micronutrient deficiencies and transfusion related iron overload might result in cardiomyopathy. There is also a tendency to develop skin cancers (squamous cell carcinoma, basal cell carcinoma and melanoma) among patients with EB. Gastrointestinal manifestation might occur in several EB subtypes and the esophagus is the most commonly affected due to repeated blistering and scarring, eventually followed by stenosis. Dysphagia, odynophagia and malnutrition belong to the most typical symptoms. They can be even exacerbated by accompanying gastroesophageal reflux disease. Pyloric atresia, painful perianal blistering and anal canal stenosis together with constipation might also occur [8–14]. In presented patient an extra-cutaneous manifestation of EB was reflected by long-lasting esophageal involvement and its stenosis. It is worth emphasizing that this patient is to our knowledge the oldest one in Poland diagnosed with EB. Nowadays an effective therapy for curing EB does not exist. No drugs are known to correct the underlying molecular defects. Even an anti-collagenase strategy, investigated in various surveys, based on phenytoin or tetracyclines, did not improve the blistering or epithelial disadhesion in EB significantly or consistently. Other recent studies focus on the suppression of transforming growth factor beta-1 (TGF-β1) and reduction of fibrosis in this mechanism. Losartan, an angiotensin II type 1 receptor antagonist was proved to down regulate TGF-β1 activators (e. g. thrombospondin 1) and to improve the course of RDEB. Immunosuppressant drugs like cyclosporine, mycophenolate mofetil and tumor necrosis alpha (TNF-α) inhibitor etanercept were also trialled, however none of them was indicated in chronic therapy of EB. Numerous potential therapies based on cell therapies (allogeneic fibroblasts, mesenchymal stromal cells, bone marrow transplantation), protein replacement and genes modification have been explored. However, further investigations must still be conducted to clarify this issue [15–18]. A modification of diet texture to soft, puree and liquids is the first step in the management of dysphagia in EB patients. If esophageal dilatation is required, endoscopic procedures are usually performed - with the use of a balloon catheter or a bougie. Nevertheless, in the most severe cases of esophageal stenosis, placement of a gastrostomy tube is recommended. Because of a broad range of complications, the treatment of EB patients must be based on a coordinated multidisciplinary approach and extend from psychological support together with dressings and padding blisters to the management of systemic complications [19–22]. Despite great advances in medicine, a targeted therapy in the course of EB has not been established yet.

## Additional file

Additional file 1: Timeline table (DOCX 15 kb)

## Abbreviations

BMI: Body mass index
DEB: Dystrophic epidermolysis bullosa
EB: Epidermolysis bullosa
EBS: Epidermolysis bullosa simplex
JEB: Junctional epidermolysis bullosa
KS: Kindler syndrome
PPI: Proton pump inhibitor
TGF-β1: Transforming growth factor beta-1
TNF-α: Tumor necrosis alpha
