# Supplementary material for: A rare case of skin blistering and esophageal stenosis in the course of epidermolysis bullosa - case report and literature review
Source: BMC Gastroenterol. 2018 Apr 13;18:47. doi: 10.1186/s12876-018-0771-5 (PMC5899341; doi:10.1186/s12876-018-0771-5)
Supplement: Supplementary file 1 — Timeline table (DOCX 15 kb) [file 12876_2018_771_MOESM1_ESM.docx]

Additional file 1

| **Date** | **Relevant Past Medical History and Interventions** | | |
| --- | --- | --- | --- |
| 1976 | Presence of single blisters on the whole body since the sixth month of life. | | |
| 1980 | Experiencing heartburn for the first time. | | |
| 1984 | Diagnosis of dystrophic epidermolysis bullosa (DEB); patient's’ sister was also diagnosed with EB | | |
| 1985 | First episodes of dysphagia. | | |
| 1995 | Deterioration of dysphagia; consumption of liquids and soft consistency meals. | | |
| 1997 | Endoscopic dilation of the esophagus (approximately 18 cm from incisors). | | |
| 1998 | Detection of esophageal constriction on the similar level (approximately 18 cm from incisors) in barium swallow test. | | |
| 2013 | Hemoptysis and a suspicion of bleeding to pulmonary alveoli in the course of DEB - excluded in a CT scan. | | |
| 2014 | Hematochezia and pain in hypogastrium; tissue samples obtained in colonoscopy revealed the presence of nonspecific inflammatory infiltration in the ascending colon and terminal part of the ileum. | | |
| 2016 | Chest pain and elevated level of troponin I; an electrocardiogram did not show any abnormalities; the patient refused to undergo coronarography and no more cardiological diagnostic procedures were performed. | | |
| **Date** | **Summaries from Initial and Follow-up** | **Diagnostic Testing (including dates)** | **Interventions** |
| Dec 2016 | Deterioration of dysphagia for two previous months | **→** A probe of gastroscopy under sedation with benzodiazepine failed due to an esophageal stenosis. An attempt of examination with paediatric endoscope was also unsuccessful.  **→** A barium swallow test revealed a narrowing of upper esophageal lumen to 7 mm along the length of 4 cm together with two diverticula on the right side not emptying of contrast. During swallowing other two diverticula appeared which were emptying of contrast.  **→** A barium swallow test also showed a noticeable weakening of the esophageal mucous membrane | **→** After the performed investigation the patient was qualified to endoscopic dilatation of esophageal stenosis and endoscopic management of diverticula. However, he did not agree to undergo this procedure during current hospital stay.  **→** The patient was treated with proton pump inhibitor (PPI) and prokinetic drugs administered intravenously, which caused an amelioration of esophageal discomfort  **→** He was discharged in a good general condition with a recommendation of a diet based on soft consistency meals, oral PPI and prokinetic drugs administration and the next follow-up in a month. |
